# Otolith tethering in the zebrafish otic vesicle requires Otogelin and α-Tectorin

**DOI:** 10.1242/dev.116632

**Published:** 2015-03-15

**Authors:** Georgina A. Stooke-Vaughan, Nikolaus D. Obholzer, Sarah Baxendale, Sean G. Megason, Tanya T. Whitfield

**Affiliations:** 1 Bateson Centre andDepartment of Biomedical Science, University of Sheffield, Sheffield S10 2TN, UK; 2 Department of Systems Biology, Harvard Medical School, 200 Longwood Avenue, Boston, MA 02115, USA

**Keywords:** Zebrafish, Otolith, Otogelin, α-Tectorin, Deafness, Vestibular disease

## Abstract

Otoliths are biomineralised structures important for balance and hearing in fish. Their counterparts in the mammalian inner ear, otoconia, have a primarily vestibular function. Otoliths and otoconia form over sensory maculae and are attached to the otolithic membrane, a gelatinous extracellular matrix that provides a physical coupling between the otolith and the underlying sensory epithelium. In this study, we have identified two proteins required for otolith tethering in the zebrafish ear, and propose that there are at least two stages to this process: seeding and maintenance. The initial seeding step, in which otolith precursor particles tether directly to the tips of hair cell kinocilia, fails to occur in the *einstein* (*eis*) mutant. The gene disrupted in *eis* is *otogelin* (*otog*); mutations in the human *OTOG* gene have recently been identified as causative for deafness and vestibular dysfunction (DFNB18B). At later larval stages, maintenance of otolith tethering to the saccular macula is dependent on *tectorin alpha* (*tecta*) function, which is disrupted in the *rolling stones* (*rst*) mutant. α-Tectorin (Tecta) is a major constituent of the tectorial membrane in the mammalian cochlea. Mutations in the human *TECTA* gene can cause either dominant (DFNA8/12) or recessive (DFNB21) forms of deafness. Our findings indicate that the composition of extracellular otic membranes is highly conserved between mammals and fish, reinforcing the view that the zebrafish is an excellent model system for the study of deafness and vestibular disease.

## Abstract (precis)

**Summary:** The tethering of zebrafish otoliths, which are important for balance and hearing in fish, occurs in two stages that are dependent on genes that have been implicated in human deafness.

## INTRODUCTION

The senses of hearing and balance depend on otoliths (ear stones) in fish. Otoliths consist of a proteinaceous core that is biomineralised by calcium carbonate; in the adult fish ear, a single otolith is tethered to each of the utricular, saccular and lagenar sensory maculae. The otoliths act as tethered masses within the ear, allowing sensation of linear accelerations and sound. By contrast, angular accelerations (turning movements) are sensed by the three semicircular canals of the inner ear; their associated sensory patches, the cristae, are not associated with otoliths. In the mammalian ear, otoconia (multiple small crystals of calcium carbonate) have equivalent vestibular functions to otoliths. The molecular mechanisms of formation and maintenance of otoliths and otoconia are likely to be conserved between fish and mammals (Hughes et al., 2006; Lundberg et al., 2014), and therefore the study of fish otolith development can provide insight into the formation of mammalian otoconia and an increased understanding of hearing and balance disorders.

Otolith formation consists of otolith seeding followed by growth by biomineralisation. In zebrafish, the utricular and saccular otoliths form from otolith precursor particles (OPPs), which are thought to consist largely of glycoproteins and may also contain glycogen (Pisam et al., 2002) and Cadherin 11 (Clendenon et al., 2009). OPPs appear in the otic vesicle (OV) at ∼18 h post fertilisation (hpf) and bind exclusively to the tips of the kinocilia of the first hair cells in the OV (also known as the tether cilia of the tether cells) (Riley et al., 1997; Tanimoto et al., 2011). There are two pairs of tether cells: one pair at the anterior pole of the OV (the presumptive utricular macula) and one pair at the posterior pole (the presumptive saccular macula). The zebrafish lagenar macula and otolith develop later, at 12-20 days post fertilisation (dpf) (Riley and Moorman, 2000; Bever and Fekete, 2002).

Adhesion of OPPs to tether cilia (otolith seeding) is thought to be mediated by one or more otolith precursor binding factors (Riley et al., 1997; Yu et al., 2011; Stooke-Vaughan et al., 2012). If tether cell differentiation is prevented by morpholino-mediated knockdown of *atoh1b*, OPPs fail to tether within the OV, and a single, untethered otolith is formed (Millimaki et al., 2007; Stooke-Vaughan et al., 2012). If tether cells are present but ciliary axonemes are absent (via genetic disruption of *dzip1* or *ift88*), OPPs tether instead directly to the apical surface of tether cells (Yu et al., 2011; Stooke-Vaughan et al., 2012). These observations support the proposal that tether cells produce one or more binding factors allowing them to act as specific tethering points for OPPs within the OV (Riley et al., 1997), but this binding factor (or factors) has not yet been identified. A number of zebrafish mutant lines show disrupted otolith formation; of these, *einstein*, *menhir* (Whitfield et al., 1996) and *monolith* (Riley and Grunwald, 1996; Riley et al., 1997), like *atoh1b* morphants, form only one otolith during early development, and so are good candidates for ear-specific components of otolith tethering.

Biomineralisation of the otoliths, through deposition of calcium carbonate, begins soon after initial seeding of the OPPs (Riley et al., 1997; Söllner et al., 2003; Yu et al., 2011; Stooke-Vaughan et al., 2012). During otolith growth, adhesion of the biomineralised otolith to the sensory patch must be maintained. This is achieved by the otolithic membrane, an acellular matrix that sits between the sensory macula and the otolith (Dunkelberger et al., 1980; Hughes et al., 2004). The otolithic membrane is equivalent to the mammalian otoconial membrane, a gelatinous matrix that supports the otoconia above the utricular and saccular epithelium in the mammalian ear.

Several glycoprotein components of the otoconial membrane have been identified in mammals, including otogelin, otogelin-like, α-tectorin, β-tectorin and otolin (Goodyear and Richardson, 2002; Deans et al., 2010; Yariz et al., 2012). The teleost otolithic membrane is thought to have a similar composition to the mammalian otoconial membrane; Otolin-1 has been identified as an otolithic membrane protein in adult rainbow trout, chum salmon and bluegill sunfish (reviewed by Hughes et al., 2006; Lundberg et al., 2006). Little is known, however, about development of the teleost otolithic membrane at embryonic stages or its composition in zebrafish. It is likely that Otolin 1a (Murayama et al., 2005), β-Tectorin (Yang et al., 2011) and Otogelin-like (Yariz et al., 2012) are components of the zebrafish otolithic membrane, based on variable phenotypes (small, fused, supernumerary or untethered otoliths) seen in morphants for these genes. Other components of the zebrafish otolithic membrane have not yet been characterised.

In this study, we have identified causative mutations for two specific otolith tethering defects in zebrafish. The disrupted genes in *einstein* and *rolling stones* mutants encode Otogelin and α-Tectorin; we identify Otogelin as a component required for seeding of OPPs and α-Tectorin as a component of the zebrafish otolithic membrane. In humans, mutations in *OTOG* and *TECTA* cause deafness and, in some cases, vestibular dysfunction, making the *einstein* and *rolling stones* zebrafish mutants new models of these disorders.

## RESULTS

### The *einstein* mutation disrupts otolith seeding

Only a single otolith forms in each ear of the zebrafish *einstein* (*eis*; German for ‘one stone’) mutant at stages when there should normally be two. This is a fully penetrant recessive phenotype, and appears to be ear specific; the mutation is homozygous adult viable. The *eis* locus was one of the largest complementation groups to be isolated in the Tübingen and Boston 1996 mutagenesis screens, with 22 alleles (Malicki et al., 1996; Whitfield et al., 1996). The ear appears otherwise to be patterned normally, but mutant embryos show vestibular dysfunction (Whitfield et al., 1996) (supplementary material Fig. S1A,B).

**Fig. 1.**
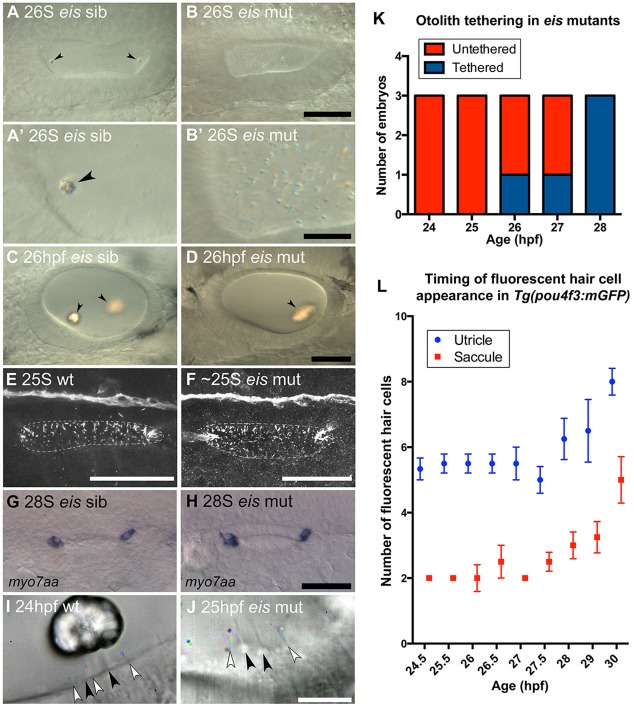
**Development of otoliths, hair cells and cilia in *eis* mutant zebrafish embryos.** (A-D) Live DIC images; lateral views with anterior to the left and dorsal up. (A) Phenotypically wild-type 26S sibling OV containing two tethered otoliths (arrowheads). (A′) Magnified view of the anterior OV pole from A. (B) 26S *eis* mutant OV. Otolith seeding has failed and there is an accumulation of OPPs in the OV. (B′) Magnified view of the anterior OV pole from B. (C) 26 hpf sibling OV. The otoliths (arrowheads) have started to biomineralise. (D) 26 hpf *eis* mutant OV. A single, large biomineralised otolith (arrowhead) has formed and is tethered above the posterior macula. (E,F) Maximum projection of confocal stacks through the entire OV of a 25S wild-type (AB strain) embryo (E) and ∼25S *eis* mutant embryo (F) stained with anti-acetylated Tubulin antibody. Dorsal view, with anterior left, lateral down. Dashed line indicates the approximate location of the OV lumen. There is no obvious difference in ciliary morphology in the *eis* mutant ear. (G) *myo7aa* mRNA expression marks tether cell pairs at the OV poles in a 28S phenotypically wild-type sibling. Dorsal view, with anterior to left. (H) *myo7aa* expression is unaffected in 28S *eis* mutant embryos. (I,J) Time-to-colour merge of six consecutive frames from movies of a 24 hpf wild-type (AB strain) OV (I; supplementary material Movie 1) and a 25 hpf *eis* mutant OV (J; supplementary material Movie 2). Dorsolateral views of left OVs are shown with anterior to right. Colour indicates movement, greyscale indicates lack of movement. Black arrowheads mark immotile kinocilia, white arrowheads mark motile cilia. (K) Tapping of *eis* mutant embryos showed that the single otolith tethers at 27-28 hpf (*n*=3 for each time point). (L) Fluorescent hair cell counts in Tg(*pou4f3:mgfp*) embryos indicate that a second wave of hair cells differentiates from 27 hpf. *n*=3 ears for 24.5 hpf; *n*=4 ears for all other ages. Error bars indicate s.e.m. Scale bars: 40 µm in A-D; 10 µm in A′,B′; 40 µm in E,F; 50 µm in G,H; 10 µm in I,J.

To understand the basis of the single otolith phenotype, we examined otolith formation in the *eis^te296f^* mutant from the earliest stages of otolith tethering. In wild-type embryos, the first signs of otolith formation are small clusters of OPPs that have seeded or tethered to the tips of the tether cell kinocilia (Fig. 1). These clusters start to form in wild-type embryos at the 18- to 19-somite (S) stage (Riley et al., 1997). At the 26S stage, two nascent otoliths are visible at the poles of the wild-type OV (Fig. 1A). By contrast, in the *eis* mutant at 26S, otoliths have not seeded and instead there is a build-up of otolith particles that remain distributed throughout the lumen (Fig. 1B). These particles are larger than the OPPs found at the earliest stages of wild-type otolith formation, and are birefringent under polarised light (Fig. 1B′), suggesting that mineralisation has begun. By 26 hpf, *eis* mutants form a single large otolith that is initially untethered and misshapen but biomineralised. This otolith adheres to one of the two sensory maculae in the ear by 28 hpf, usually the posterior macula (Fig. 1D).

The early failure of otolith seeding in *eis* mutants is reminiscent of the phenotype of *atoh1b* morphants, in which the first sensory hair cells (tether cells) fail to form (Millimaki et al., 2007; Stooke-Vaughan et al., 2012). To test whether hair cell or ciliary defects might underlie the failure of otolith formation in *eis*, we examined hair cells, cilia and ciliary motility in the *eis* mutant ear. In the early *eis* mutant OV (25S), as in the wild type, groups of longer cilia were present at the anterior and posterior poles, as determined by anti-acetylated tubulin staining, which also labels the tether cells (Fig. 1E,F). The presence of tether cells was also confirmed by *myo7aa* expression; no difference was seen between *eis* mutant and sibling embryos (Fig. 1G,H). High-resolution video microscopy at 25 hpf revealed that the otic cilia included both immotile sensory hair cell kinocilia and motile cilia on neighbouring cells, as in the wild type (Fig. 1I,J; supplementary material Movies 1and 2). These data indicate that the failure in otolith seeding in *eis* mutants cannot be attributed to a loss of hair cells, kinocilia or ciliary motility.

To determine the stage at which the *eis* otolith became tethered, we photographed the ear in mutant embryos to record otolith position, tapped the slide on which they were mounted on the benchtop, and then rephotographed the ear. This tapping never disrupted otolith position in wild-type embryos (*n*=10; data not shown). However, in *eis* mutants, the otolith could be dislodged in this way up until 27 hpf (*n*=10 of 12 *eis* mutants tested between 24 and 27 hpf; Fig. 1K). After this stage, the single otolith became attached, usually to the posterior (saccular) macula. The time at which the single otolith in *eis* mutants adheres to the posterior macula corresponds to the time at which a second wave of hair cells differentiates (Fig. 1L), implying that there is a mechanism for tethering the biomineralised otolith that might require the second wave of hair cells. This second wave of hair cells is known to be dependent on *atoh1a* function (Millimaki et al., 2007). To test whether these *atoh1a-*dependent hair cells are required to tether the biomineralised otolith in *eis*, we injected *eis* mutant embryos with an *atoh1a* morpholino to block production of the second wave of hair cells, together with a *p53* (*tp53* – ZFIN) morpholino to reduce non-specific morpholino toxicity. The *atoh1a* morphant *eis* embryos formed a single otolith, which had tethered in only 1 of 4 embryos by 30 hpf, but was tethered in 4 of 4 embryos by 34 hpf (supplementary material Fig. S2). Despite co-injection of the *p53* morpholino, embryos still showed delayed development, so it seems likely that tethering of the single otolith in *eis* mutants does not require expression of *atoh1a* or differentiation of further hair cells. We propose that the production of a different tethering factor, independent of *eis* or *atoh1a* function, is required for tethering the single otolith in *eis* mutants. A candidate for such a factor is α-Tectorin (see below).

### The gene disrupted in the *eis* mutant is *otogelin*

**Fig. 2.**
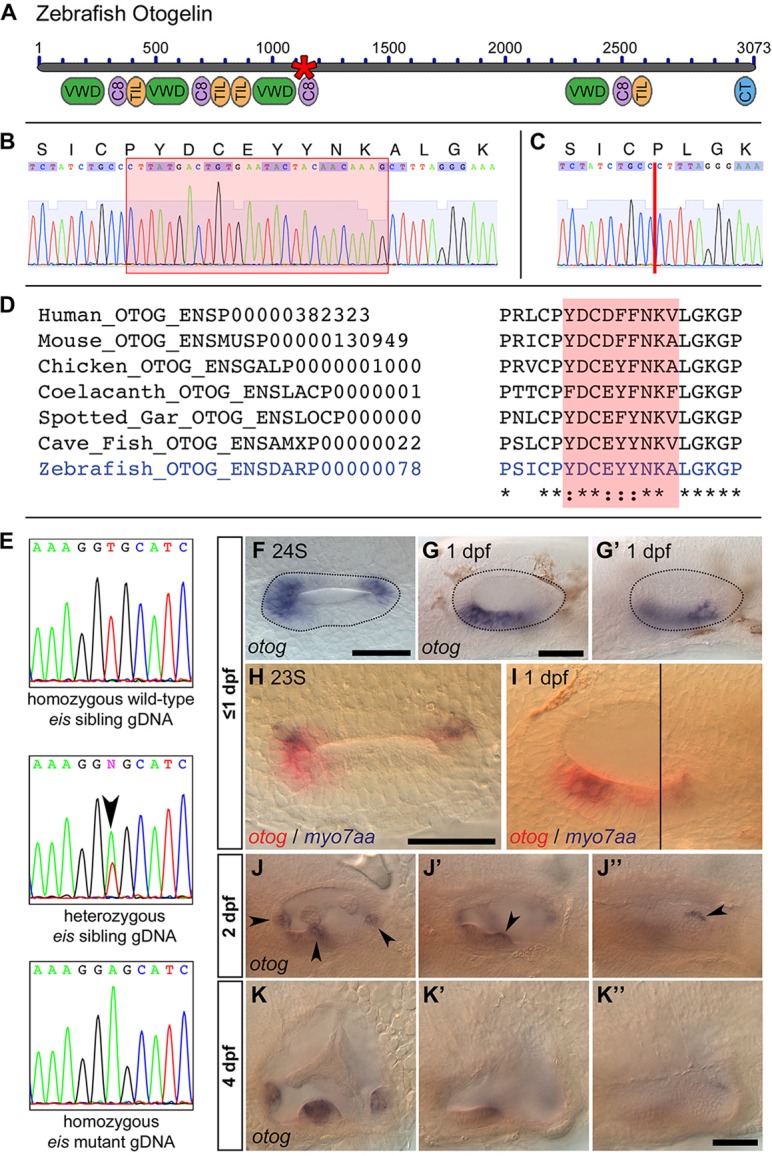
**The *eis* mutation corresponds to a lesion in *otog.*** (A) Overview of Otogelin protein structure based on available sequence data; the N terminus is likely to be incomplete. The asterisk indicates the predicted in-frame deletion in the *eis^te296f^* allele. VWD, Von Willebrand factor type D domain; C8, domain containing eight conserved cysteine residues; TIL, trypsin inhibitor-like cysteine-rich domain; CT, C-terminal cysteine knot-like domain. (B) Pooled wild-type (strain LWT) cDNA sequence data and predicted amino acid translation covering the region of *otog* that is mutated in *eis^te296f^*. The red box indicates the sequence deleted in *eis^te296f^*. (C) Pooled *eis^te296f^* cDNA from the same region as shown in B. The deletion is indicated by a vertical red line. (D) Clustal 2.1 multiple sequence alignment of the region of Otogelin deleted in the *eis^te296f^* allele. The nine amino acids deleted in *eis^te296f^* are highly conserved across vertebrates (shading). (E) gDNA sequence data from wild-type sibling, *eis^te296f^* heterozygous sibling and homozygous mutant embryos, confirming the T-to-A transversion identified by HMFseq (arrowhead indicates the double peak in the heterozygous embryo). (F-G′) Dorsal (F, 24S) and lateral (G,G′, 1 dpf) views of a wild-type OV (dotted outline), showing that *otog* mRNA expression is not restricted to the tether cells. (G,G′) Different focal planes of the same OV. Anterior is to left. (H) Dorsal view of 23S wild-type OV; anterior to left. Expression of *otog* mRNA (red) includes the tether cells (marked by *myo7aa*, blue) and other cells at the poles of the OV. (I) Lateral view of 1 dpf wild-type OV. Two focal planes are combined (black line marks the join), showing the anterior macula (left) and the posterior macula (right). *otog* (red) is expressed in hair cells (*myo7aa* positive, blue) and surrounding epithelial cells. (J-J″) Lateral view of different focal planes of the same 2 dpf wild-type OV, showing expression of *otog* in the cristae (J, arrowheads), at the posterior of the anterior macula (J′, arrowhead) and along the dorsal edge of the posterior macula (J″, arrowhead). (K-K″) At 4 dpf, *otog* is still expressed in the cristae, but expression is now very weak in the maculae (the apparent macula stain in K′ is out-of-focus staining in the lateral crista). Scale bars: 50 µm.

The *eis^tt272b^* allele had previously been rough-mapped to linkage group 7 (Geisler et al., 2007). We used whole-genome sequencing-based homozygosity mapping and bioinformatic filtering of pooled mutants (HMFseq) (Obholzer et al., 2012) to confirm the location on linkage group 7 for the *eis^te296f^* allele (supplementary material Fig. S3). This approach identified one candidate single nucleotide polymorphism (SNP) in the *otogelin* (*otog*) gene, affecting the splice donor site of intron 28-29. cDNA from pooled *eis* mutant embryos was used for amplification of the region of interest by standard PCR. The splice donor site SNP results in the deletion of exon 28, producing an in-frame deletion of the nine amino acid sequence YDCEYYNKA from the D3 domain of the Otogelin protein (Fig. 2A,C). This sequence is not repeated elsewhere in the protein and is highly conserved across vertebrates (Fig. 2D). Sequencing of genomic DNA (gDNA) from individual wild-type (*n*=2), mutant (*n*=2) and sibling (*n*=6: one homozygous wild type, five heterozygous) embryos from a cross between *eis* heterozygous parents confirmed genetic linkage of the SNP identified by HMFseq with the *eis* locus (Fig. 2E).

### Expression pattern of *otog* mRNA in the developing ear

Given the similarity of the phenotype to that of the *atoh1b* morphant, which lacks tether cells, we expected the expression of *otog* mRNA to be hair cell specific. However, early expression of *otog* marked two broad domains at the OV poles; later, expression appeared in a region of ventral epithelium between them (Fig. 2F-G′). Early expression appeared to encompass all cells in the presumptive sensory epithelia and was not restricted to tether cells (Fig. 2H,I). We did not detect expression elsewhere in the embryo, apart from transient expression at 1 dpf in the area of the trigeminal placode (data not shown). At later stages (2-4 dpf), *otog* was expressed in the cristae of the developing ear, whereas expression in the maculae decreased, but persisted at the dorsal edge of the saccular macula (Fig. 2J-K″). Expression of *otog* was unaffected in *eis^te296f^* mutant embryos (data not shown).

### The *rolling stones* mutation results in a loss of otolith tethering during larval stages

**Fig. 3.**
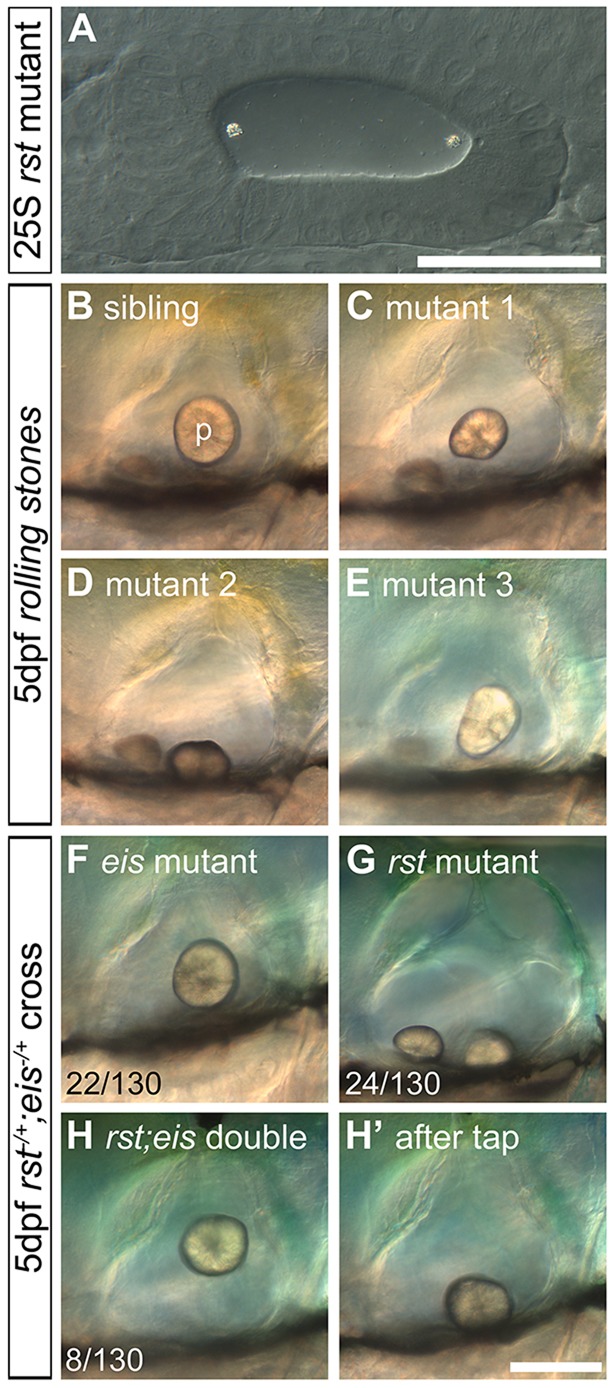
**Development of otoliths in *rst* mutant and *rst;eis* double-mutant embryos.** Live DIC micrographs of sibling and *rst* mutant OVs. The left OV is shown, with anterior to the left and dorsal to the top. (A) 25S embryo from a homozygous mutant *rst* cross. The otoliths seed normally at the anterior and posterior poles (compare with Fig. 1A). (B) 5 dpf phenotypically wild-type sibling OV. p, posterior otolith. (C) 5 dpf *rst* mutant OV. The posterior otolith is misshapen, detached and free to move. (D) OV of a different *rst* mutant, with a posterior otolith that has become detached and stuck on the ventral floor of the OV. (E) OV of an *rst* mutant with the most common phenotype of a misshapen posterior otolith. (F) 5 dpf *eis* mutant with a single otolith tethered to the posterior macula. (G) 5 dpf *rst* mutant with an untethered posterior otolith. (H) 5 dpf *rst;eis* double mutant with a single untethered otolith. (H′) The same embryo as in H, after the slide was tapped to displace the untethered otolith. Numbers indicate embryos showing each phenotype (detached or misshapen posterior otolith) among total examined (130) from an *rst;eis* double-heterozygote cross; the remaining 76/130 embryos were phenotypically wild type (not shown). Scale bars: 50 µm in A; 100 µm in B-H′.

**Fig. 6.**
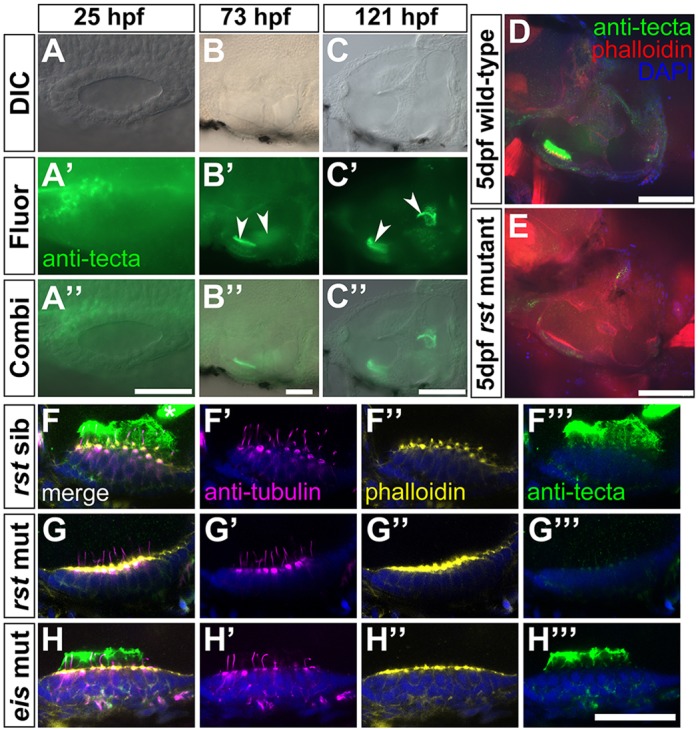
**Expression of α-Tectorin protein in wild-type and *rst* mutant embryos.** (A-C″) Immunofluorescence analysis showing that α-Tectorin protein is localised to the anterior OV at 1 dpf and to the two otolithic membranes at 3 and 5 dpf (arrowheads). Anterior is to the left. (D) Confocal image showing α-Tectorin protein localisation to the utricular otolithic membrane and to cells in the utricular epithelium (green) in a 5 dpf wild-type (AB strain) embryo. Anterior left, dorsal up. (E) In a 5 dpf *rst* embryo, α-Tectorin is not localised to the otolithic membrane of the utricular macula, although there is a low level of protein detectable within the utricular epithelium. (F-H‴) Confocal images of utricular maculae from 5 dpf embryos; lateral views, anterior to left. Nuclei are stained with DAPI (blue), hair cells and kinocilia with anti-acetylated Tubulin antibody (magenta), filamentous actin and hair cell stereociliary bundles with Alexa647-phalloidin (yellow), and α-Tectorin by antibody (green). (F-F‴) Phenotypically wild-type 5 dpf *rst* sibling embryo, showing strong staining for α-Tectorin in the utricular otolithic membrane, and protrusion of the hair cell kinocilia into this membrane. α-Tectorin staining is also visible in the saccular otolithic membrane (asterisk, F). (G-G‴) 5 dpf *rst* mutant embryo with no extracellular α-Tectorin stain. There is a weak α-Tectorin signal at the apical surface of the hair cells (G‴). Kinocilia appear normal (G′). (H-H‴) 5 dpf *eis* mutant embryo with normal expression and localisation of α-Tectorin and normal kinocilia. Scale bars: 50 µm in A-B″; 100 µm in C-E; 20 µm in F-H‴.

We also examined otolith formation in a second mutant, *rolling stones* (*rst*), isolated (as a single allele) in the Tübingen 1996 screen (Whitfield et al., 1996). As its name suggests, the *rst* mutant has otoliths that are loose within the ear. The *rst* phenotype appears to be completely ear specific; the mutation is homozygous adult viable. Mutants are indistinguishable from siblings during initial stages of otolith tethering and formation (1-2 dpf; Fig. 3A, compare with Fig. 1A). At 3-5 dpf, embryos showed a range of phenotypes: a misplaced saccular otolith (*n*=33/110 *rst* mutant embryos) that was either untethered and free to move within the OV (Fig. 3C) or stuck on the ventral floor of the OV (Fig. 3D); or a saccular otolith in the correct location within the OV but subtly misshapen or misoriented (*n*=77/110 *rst* mutant embryos; Fig. 3E). The utricular otolith did not appear to be affected; consistent with this, vestibular deficits were not apparent in *rst* mutant larvae (supplementary material Fig. S1C,D). The biomineralisation of both otoliths and the development of the OV, including formation of semicircular canal pillars (supplementary material Fig. S4), hair cells and kinocilia (see Fig. 6), appeared to occur normally.

### Double *rst;eis* mutants generate a single otolith that never becomes tethered

Our results suggest that otolith tethering occurs in at least two stages: an early seeding stage, dependent on Otogelin, in which OPPs tether to kinociliary tips; and a later maintenance stage, which is disrupted in the *rst* mutant, that is required for continued tethering of the biomineralised otolith to the saccular sensory macula. We therefore predicted that in an *rst;eis* double mutant, in which both stages of otolith tethering are disrupted, adhesion of the otoliths to the developing maculae should fail altogether. We recovered double-homozygous mutants from an *rst*^+/−^*;eis*^+/−^ incross at the expected 1:16 ratio for two independently segregating Mendelian mutations (*n*=8/130). As predicted, the double-mutant phenotype was additive. At early stages, otolith seeding to kinociliary tips was disrupted, as in *eis* single mutants (data not shown). A single otolith eventually formed but, unlike in *eis* single mutants, it never became tethered to a sensory macula, and tapping experiments showed that it was untethered until at least 5 dpf (Fig. 3H,H′). The morphology of the ear appeared otherwise normal in the double mutants (Fig. 3H; data not shown).

### The gene disrupted in the *rst* mutant is *tectorin alpha*

**Fig. 4.**
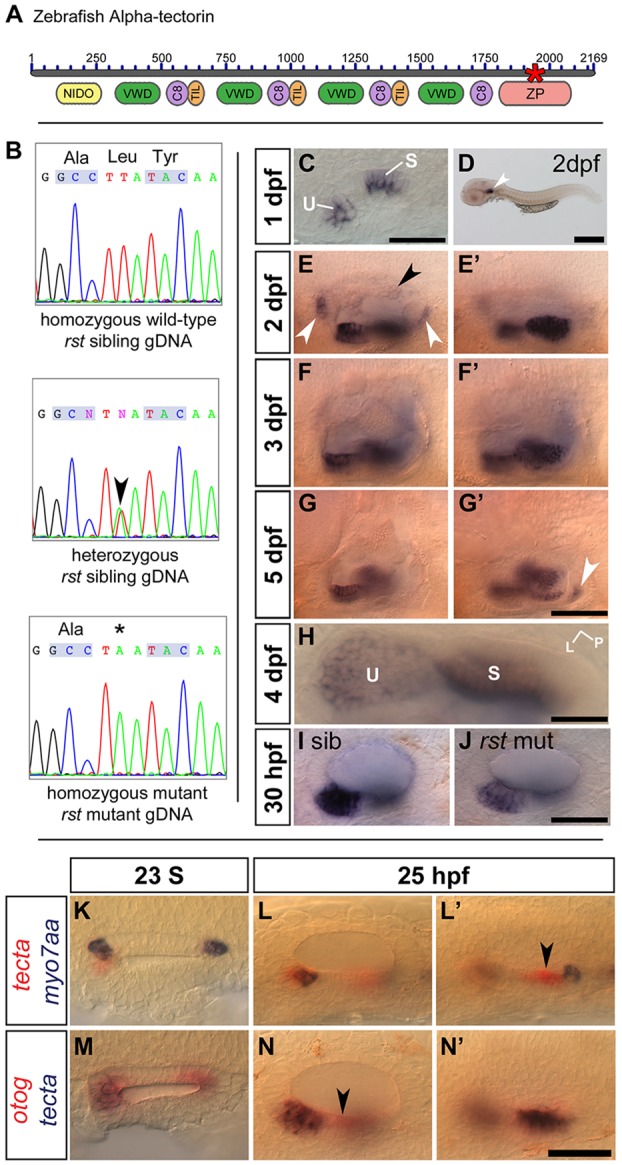
**The *rst* mutation corresponds to a lesion in *tecta.*** (A) Overview of α-Tectorin protein structure based on available sequence data; the N terminus is likely to be incomplete. The asterisk shows the location of the predicted truncation in the *rst^tl20e^* allele. NIDO, nidogen domain; VWD, Von Willebrand factor type D domain; C8, domain containing eight conserved cysteine residues; TIL, trypsin inhibitor-like cysteine-rich domain; ZP, zona pellucida domain. (B) Sequencing data from *rst* mutant, heterozygous sibling and homozygous wild-type sibling embryos showing the T-to-A transversion (arrowhead and asterisk). (C) *tecta* mRNA expression in the OV of a 1 dpf phenotypically wild-type sibling embryo from an *eis* cross. *tecta* is expressed in the utricular (U) and saccular (S) maculae. (D) 2 dpf wild-type (AB strain) embryo showing that *tecta* is expressed exclusively in the OV (arrowhead). (E,E′) Within the OV at 2 dpf, *tecta* is expressed at high levels in the utricle and saccule, with lower levels in the semicircular canal projections (black arrowhead) and at the base of the cristae (white arrowheads). (F,F′) Strong *tecta* expression continues in the utricular and saccular maculae at 3 dpf. (G,G′) The level of *tecta* expression in the maculae is reduced at 5 dpf, and a new region of expression appears (arrowhead). (H) Dorsal view of utricle (U) and saccule (S) in 4 dpf wild-type embryo. L, lateral; P, posterior. Expression in the utricular macula is strongest at the periphery. (I,J) *rst* mutants show a lower level of *tecta* expression than siblings at 30 hpf. Genotypes were confirmed by sequencing gDNA. (K) Dorsal view of a 23S wild-type OV, showing that *tecta* mRNA expression (red) is not restricted to the tether cells (*myo7aa* mRNA, blue) at seeding stages. (L,L′) Lateral views of 25 hpf phenotypically wild-type OV. The focal plane shows the region of the anterior macula in L and the region of the posterior macula in L′. *tecta* expression includes the tether cells and surrounding cells of the anterior macula (L) and a region just anterior to the tether cells of the posterior macula (arrowhead, L′). (M) Dorsal view of a 23S wild-type OV, showing that *otog* (red) and *tecta* (blue) expression roughly overlap at this stage. (N,N′) Lateral views of a 25 hpf phenotypically wild-type OV. The focal plane shows the region of the anterior macula in N and the region of the posterior macula in N′. *otog* (red) is expressed in a *tecta*-negative area of the ventral OV epithelium (arrowhead, N). Scale bar: 50 µm in C,I,J,K-N′; 500 µm in D; 33 µm in H; 75 µm in E-G′.

The *rst^tl20e^* mutation was rough-mapped to linkage group 5 (Geisler et al., 2007), and we noted that an orthologue of the human gene tectorin alpha (*TECTA*) is present in the critical region. As α-Tectorin is a known component of the otoconial membrane in mammals (Goodyear and Richardson, 2002), we set out to test zebrafish *tecta* as a candidate for *rst*. Sequencing of *tecta* cDNA from *rst* mutants revealed a T-to-A transversion mutation in exon 24, resulting in a premature stop codon in the zona pellucida (ZP) domain of the protein (Fig. 4A,B). This mutation was not present in wild-type embryos (AB strain; *n*=2). Sequencing of gDNA from individual mutant and sibling embryos confirmed the presence of the mutation in all *rst* mutant embryos and heterozygote siblings and its absence from wild-type siblings (*n*=28: nine wild type, ten heterozygotes and nine homozygous mutants).

### Expression of *tecta* is specific to sensory maculae in the zebrafish ear and is reduced in the *rst* mutant

**Fig. 5.**
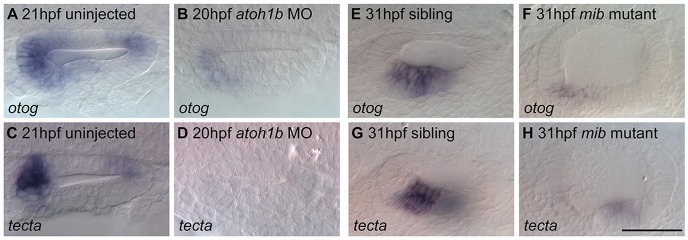
**Normal macular development is required for normal expression of *otog* and *tecta.*** (A) *otog* mRNA expression at the poles of the OV of a 21 hpf wild-type (LWT strain) OV. (B) Expression of *otog* was reduced in the OV of *atoh1b* morphants. (C) Expression of *tecta* at the poles of a 21 hpf wild-type OV. (D) *tecta* expression was not detected in the OV of *atoh1b* morphants. (E) *otog* expression in the utricular macula of a 31 hpf phenotypically wild-type sibling embryo. (F) *otog* expression was reduced in the utricular macula of a 31 hpf *mib* mutant embryo. (G) *tecta* expression in the utricular macula of a 31 hpf phenotypically wild-type sibling embryo. (H) *tecta* expression was reduced in the utricular macula of a 31 hpf *mib* mutant embryo. Weak expression remained in the saccular macula (out of focus). Dorsal (A-D) and lateral (E-H) views, with anterior to left. Scale bar: 50 µm for A-H.

The *tecta* transcript specifically marks the developing sensory maculae in the zebrafish ear (Fig. 4C-H), corresponding to the observed protein expression (see below). As in the mouse (Goodyear and Richardson, 2002), zebrafish *tecta* is not expressed in the cristae (Fig. 4F,G). *tecta* is expressed equally strongly in both maculae, and so the differential adhesion of the otoliths to the two sensory patches in the *rst* mutant is likely to depend on an additional factor or factors. At 2 dpf, *tecta* is also expressed at the base of the developing cristae and in the semicircular canal projections (Fig. 4E). As the sensory patches grow, expression of *tecta* becomes strongest at the edges of the sensory maculae (Fig. 4H). In the *rst* mutant, *tecta* RNA expression was present, but was weaker than normal at 30 hpf (Fig. 4I,J) and 5 dpf (not shown), possibly owing to nonsense-mediated decay of the mutant transcript. At early stages of otolith formation, *tecta* expression is not limited to the tether cells (Fig. 4K-L′). *tecta* and *otog* are expressed in similar regions of the OV at 23S (Fig. 4M), but *tecta* is never expressed in the ventral epithelium of the OV, in contrast to *otog* (Fig. 4N). Expression of *tecta* was unaffected in *eis* embryos, and expression of *otog* was unaffected in *rst* embryos from 1-5 dpf (data not shown).

### Normal macular development is required for normal expression of *otog* and *tecta*

From our *in situ* hybridisation analysis, expression of *otog* and *tecta* appeared to mark both hair cells and supporting cells in the sensory maculae, and was not restricted to the tether cells at 21 hpf (Fig. 2, Fig. 4 and Fig. 5A,C). To determine whether specification of tether and supporting cells is required for *otog* and *tecta* expression, we examined expression in embryos injected with a morpholino against *atoh1b*, which do not form tether cells and are also likely to lack the first supporting cells (Woods et al., 2004; Millimaki et al., 2007). *atoh1b* morphants showed a loss of expression of *otog* and *tecta* at stages corresponding to initial otolith seeding and growth (Fig. 5B,D). This suggested that normal *atoh1b*-dependent early macular development is required for early expression of *otog* and *tecta*.

At later stages of macular development, expression of both genes still appeared to span both the hair cell and supporting cell layers of the sensory epithelium in wild-type embryos. To determine whether hair cells or supporting cells were required for expression of *otog* and *tecta* at this later stage, we examined their expression in the *mib1* mutant *mib^ta52b^*, which develops supernumerary hair cells at the expense of supporting cells in the ear due to disrupted Notch signalling (Haddon et al., 1998, 1999). If *otog* and *tecta* were expressed in hair cells, then we expected an upregulation of expression in the *mib* mutant ear, whereas if they were expressed in supporting cells, then we expected a loss of expression. We found that expression of *otog* and *tecta* was severely downregulated in *mib* mutant embryos at 31 hpf (Fig. 5F,H), suggesting that *otog* and *tecta* expression in the wild-type sensory patch at this stage requires the presence of supporting cells.

### Expression pattern of α-Tectorin protein in the ear

To examine whether there was a lack of α-Tectorin protein in the *rst* mutant, we used a polyclonal antiserum raised against the von Willebrand domain (VWD) repeats of the chick α-tectorin protein (Knipper et al., 2001). In wild-type embryos, this antiserum stained a region anteromedial to the OV at 1 dpf (*n*=6) (Fig. 6A-A″), and the otolithic membranes over the utricular and saccular maculae at 3 dpf (*n*=6) and 5 dpf (*n*=6) (Fig. 6B-C″). In the *rst* mutant, protein expression was reduced in the sensory patches and undetectable in the otolithic membrane (Fig. 6E). Since the *rst* mutation predicts a deletion of the ZP domain downstream of the VWD repeats, the antiserum would be expected to cross-react with the truncated protein, if produced. In phenotypically wild-type embryos, the hair cell kinocilia protrude into the otolithic membrane (Fig. 6F). In *rst* mutant embryos, a very weak signal was detected within the utricular macular epithelium, but none was detected in the extracellular space above the hair cells, where the otolithic membrane should be (Fig. 6E,G″). This result indicates that very little mutant protein is produced and, if present, is unable to assemble within the otolithic membrane. Otogelin function does not appear to be required for the normal assembly of α-Tectorin into the otolithic membrane, as α-Tectorin staining appeared normal in *eis* mutants, at least at these stages (Fig. 6H). Taken together with the sequencing data, these results strongly suggest that the gene disrupted in *rst* is *tecta*.

## DISCUSSION

### Otolith tethering and adhesion in the zebrafish embryo

Our data support a two-step model of otolith tethering in the zebrafish ear. The first step involves OPP tethering to the tips of the tether cell kinocilia, defined by Riley and colleagues as otolith seeding, which occurs at 18-22 hpf (Riley et al., 1997). Otogelin is required for this seeding step: without it, OPPs fail to adhere to the tether cilia. However, we found that *otog* mRNA expression was not tether cell specific at otolith seeding stages in zebrafish. As otogelin is known to be secreted into the lumen of the ear in mouse (Cohen-Salmon et al., 1997), we suggest that it is also secreted into the lumen of the OV in zebrafish, where it might interact with a tether cell-specific binding factor that is localised to the tips of the tether cilia. Otogelin might be a component of OPPs, enabling them to recognise the tether cilia as exclusive tethering points within the OV. Alternatively, Otogelin might be bound to the tips of the tether cilia by a membrane-bound protein, such as the hair cell-specific integrin α8β1 (Littlewood Evans and Müller, 2000); the resultant complex could then be capable of tethering the OPPs. In order to test these hypotheses, it will be important to determine the localisation of Otogelin within the OV.

It is likely that the chaperone protein Hsp90β1 acts upstream of Otogelin, where it might be involved in the processing and secretion of Otogelin and other OPP or matrix components. *hsp90b1* mRNA is expressed throughout the medial wall of the OV epithelium at 20S (Sumanas et al., 2003), in a wider expression domain than that of *otog* at 24S. The *monolith* mutant and *hsp90b1* morphants, like *eis* mutants, display disrupted otolith seeding followed by the formation of a single posterior otolith (Riley and Grunwald, 1996; Riley et al., 1997; Sumanas et al., 2003); *monolith* has recently been shown to be due to a mutation in *hsp90b1* (B. Riley, personal communication).

Other proteins that might contribute to normal otolith seeding in the zebrafish embryo include Otoconin 90 (Oc90, previously Otoc1), Sparc and Otolith matrix protein (Otomp). *oc90* morphant embryos display a range of otolith phenotypes, suggesting defects in the seeding of OPPs (Petko et al., 2008). Oc90 is the major organic component of mammalian otoconia (Wang et al., 1998), and so is likely to be a component of OPPs in zebrafish. Zebrafish *sparc* morphants also show a variety of otolith defects, including an abnormal number, small, fused or absent otoliths (Kang et al., 2008). Otomp is expressed from early otic placode stages; *otomp* morphant embryos show no apparent defect in OPP seeding but have slowed otolith growth (Murayama et al., 2005). A tether cell-specific ‘OPP binding factor’ therefore remains elusive: no gene has yet been identified that disrupts otolith seeding and is expressed exclusively in the tether cells.

The second step of otolith formation is the maintenance of otolith adhesion to the sensory maculae during growth of both the biomineralised otoliths and the maculae. For the posterior (saccular) otolith, we have shown this requires α-Tectorin function, which is disrupted in the *rst* mutant. Our antibody data for the α-Tectorin protein demonstrate, for the first time, that extracellular otolithic membranes have formed in the zebrafish ear by 72 hpf. Within these membranes, α-Tectorin is likely to interact with organic matrix components of the otolith to maintain adhesion of the otolith to the macula. Otolin 1a has been shown to be a component of chum salmon and rainbow trout otoliths and otolithic membrane (Murayama et al., 2002, 2004) and of zebrafish otoliths (Murayama et al., 2005). An interaction between the nidogen domain of α-Tectorin and the collagenous domain of Otolin 1 has been proposed previously (Lundberg et al., 2006). Expression of *otolin 1a* (*otol1a*) is not detected until 48 hpf in the zebrafish embryo, but it is expressed strongly at the dorsal and ventral edges of the saccule at 72 hpf; no expression was detected in the utricle (Murayama et al., 2005). Zebrafish *otol1a* morphants show a variable phenotype, but in some cases the otoliths become only loosely associated with their corresponding maculae, eventually fusing into a single otolith (Murayama et al., 2005). β-Tectorin consists of an isolated ZP domain, and zebrafish *tectorin beta* morphants show a similar otolith phenotype to *otol1a* morphant embryos (Yang et al., 2011). Zebrafish *otogelin-like* (*otogl*) morphants show a range of ear and other defects, including a small saccular otolith (Yariz et al., 2012). It therefore seems likely that α-Tectorin, β-Tectorin, Otolin 1a, Otogelin-like and Otogelin are all components of the acellular otolithic membranes in zebrafish.

### Relevance for human disease

Mutations in the human orthologues of both *otog* and *tecta* are associated with disease. Mutations in *OTOG* have recently been identified as causative for autosomal recessive non-syndromic deafness with vestibular deficits, designated DFNB18B (Schraders et al., 2012; Oonk et al., 2014). Clinical features can include delayed motor development, suggesting early onset vestibular dysfunction, and vestibular hyporeflexia in teenage years. Mice mutant for *Otog* are deaf and display a severe vestibular phenotype (Simmler et al., 2000a,b), consistent with the location of otogelin protein in all acellular membranes of the inner ear (Cohen-Salmon et al., 1997). In *Otog*^−/−^ mice, otolithic membranes and their attached otoconia are displaced from postnatal day 2 onwards (Simmler et al., 2000b); this differs from our observations in 5 dpf zebrafish, in which the α-Tectorin-positive otolithic membrane remained attached to the macula in the *eis* mutant. Our results suggest a specific early role for zebrafish Otogelin in tethering OPPs to kinociliary tips before biomineralisation.

α-Tectorin is abundant in the tectorial membrane of the mammalian cochlea, which does not have a direct counterpart in the fish. Mutations in the human *TECTA* gene cover every domain of the protein, and result in both autosomal dominant [DFNA8/12; Online Mendelian Inheritance in Man (OMIM) #601543] and autosomal recessive (DFNB21; OMIM #603629) non-syndromic hearing loss. Where tested, vestibular function is often normal, but there are occasional reports of vestibular hyporeflexia or vertigo (Li et al., 2013; Ishikawa et al., 2014). Several missense mutations have been identified within the ZP domain; these all result in mid-frequency hearing loss, which may be stable or progressive (Hildebrand et al., 2011; and references therein). In the zebrafish *rst* (*tecta*) mutant, which predicts a deletion of over half the ZP domain, very little protein is detectable in the mutant ear, and it is unlikely that any protein that is produced would be able to assemble correctly in the otolithic membrane.

Several mouse models carrying mutations in *Tecta* have been generated (Legan et al., 2014; and references therein). The effects of different *Tecta* mutations on the tectorial membrane have been analysed in detail, but less is known about Tecta function in the mammalian vestibular system, where it is also expressed (Rau et al., 1999; Goodyear and Richardson, 2002). Mice lacking *Tecta* function have reduced otoconial membranes, with fewer and larger otoconia, but no obvious vestibular behavioural deficits (Legan et al., 2000). Our findings in the *rst* mutant are the first to suggest a specific role for α-Tectorin in the maintenance of otolith tethering.

More generally, an understanding of the composition and function of the otolithic or otoconial membranes will be of relevance for other vestibular disorders. Dizziness in the elderly is common and may be related to a loss of vestibular hair cells, demineralisation of otoconia or degeneration of the otoconial membrane (Andrade et al., 2012; and references therein). Benign paroxysmal positional vertigo (BPPV), in which otoconia become detached and lodge in one of the semicircular canals (canalithiasis) or cupulae (cupulolithiasis), is also a relatively common disorder. In many cases the primary cause is head trauma, but other cases are idiopathic; familial incidence, suggesting genetic predisposition, and increasing prevalence in the elderly have been reported. Genes that encode components of the otoconial membrane, such as *OTOG* or *TECTA*, might be good candidates for genetic predisposition to this disorder or for understanding age-related vestibular dysfunction (Hughes et al., 2004; Deans et al., 2010). The zebrafish mutants described here will be a useful addition to the model systems available to study vestibular disorders associated with otoconial abnormality.

## MATERIALS AND METHODS

### Ethics statement

All animal experiments conformed to UK Home Office regulations.

### Animals

Zebrafish (*Danio rerio*) wild-type lines used were AB and London Wild Type (LWT); mutant lines were *eis^te296f^*, *mib^ta52b^* and *rst^tl20e^* (Jiang et al., 1996; Whitfield et al., 1996). All mutant embryos were homozygous for the zygotic mutant allele. ‘Siblings’ refers to stage-matched, phenotypically wild-type embryos from a cross between heterozygous carriers. Hair cell counts were made in the Tg(*pou4f3:mgfp*) line (Xiao et al., 2005). Embryos were raised in E3 medium (5 mM NaCl, 0.17 mM KCl, 0.33 mM CaCl_2_, 0.33 mM MgSO_4_, 0.0001% Methylene Blue). Embryonic stages are given as hours or days post fertilisation (hpf or dpf) at 28.5°C or as somite stage (S) (Westerfield, 2000). For behavioural analysis, see the supplementary material Methods.

### Megamapping the *eis* mutant

Genomic DNA was isolated from 30 pooled 3 dpf *eis^te296f^* embryos using standard methods (Westerfield, 2000). Next-generation sequencing (NGS) library preparation was performed with the NEBNext DNA Library Prep Kit (New England BioLabs) according to the manufacturer's specifications (average insert size 250 bp). The library was sequenced on a single Illumina HiSeq 2000 lane in paired-end mode with 50 bp read length. Data were analysed using the MegaMapper pipeline as described (Obholzer et al., 2012); for further details, see the supplementary material Methods.

### Positional cloning of the *rst* mutant

Total RNA was extracted from zebrafish embryos using TRIzol (Invitrogen) and converted to cDNA using the Superscript III Kit (Invitrogen) with oligo(dT) primers. A *tecta* clone covering exons 2-27 of Ensembl transcript ENSDART00000082896 was cloned into the pGEM-T Easy (Promega A1360) vector. Primer sequences are listed in Table S1 in the supplementary material.

### Immunohistochemistry and phalloidin staining

Antibody and phalloidin (Alexa 593 and Alexa 647, Invitrogen) staining were performed as described (Haddon and Lewis, 1996). Primary antibodies: mouse monoclonal anti-acetylated Tubulin (Sigma T6793, 1:100) and rabbit polyclonal antisera raised against the VWD repeats of chick α-Tectorin [(Knipper et al., 2001), 1:500]. Secondary antibodies: anti-mouse TRITC (Sigma, T5393; 1:50) and anti-rabbit FITC (Sigma, F9887; 1:200).

### *In situ* hybridisation

To generate templates for *in situ* hybridisation probes, part of the *otog* cDNA covering exons 35-42/43 was cloned into the pCRII vector (Life Technologies K2070) (for primer sequences, see Table S1 in the supplementary material). The *tecta* clone covering exons 2-27 described above was used for the *tecta* template. The *myo7aa* probe has been described previously (Ernest et al., 2000). Single and double *in situ* hybridisation were performed as described (Oxtoby and Jowett, 1993; Nüsslein-Volhard and Dahm, 2002).

### Morpholino injection

The *atoh1b* morpholino was injected as described (Stooke-Vaughan et al., 2012). For *atoh1a* and *p53* morpholino co-injections, 1- to 4-cell embryos were co-injected with 4 ng *atoh1a* morpholino (Millimaki et al., 2007) and 6 ng *p53* morpholino (Robu et al., 2007). Injection of *atoh1a* morpholino alone resulted in widespread non-specific cell death (data not shown).

### Microscopy

Live and stained embryos were photographed on an Olympus BX51 compound microscope equipped with DIC optics, using a Camedia C-3030ZOOM camera and CELL-B software (Olympus). High-speed video microscopy was undertaken and time-to-colour merges of movies were made as described (Stooke-Vaughan et al., 2012). Fluorescent samples were imaged on a laser-scanning confocal microscope (Leica SP1 or Nikon A1) or a spinning disc confocal system (PerkinElmer Ultraview Vox with an Olympus IX81 microscope). Images were assembled using Adobe Photoshop and Fiji (ImageJ) (Schindelin et al., 2012).

## Supplementary Material

Supplementary Material
