# Supplementary material for: Otolith tethering in the zebrafish otic vesicle requires Otogelin and α-Tectorin
Source: Development. 2015 Mar 15;142(6):1137–45. doi: 10.1242/dev.116632 (PMC4360185; doi:10.1242/dev.116632)
Supplement: Supplementary Material [file supp_142_6_1137__index.html]

Supplementary Material 

# Otolith tethering in the zebrafish otic vesicle requires Otogelin and α-Tectorin

## DEV116632 Supplementary Material

**Files in this Data Supplement:**

- Supplementary Material
