# Supplementary material for: Otolith tethering in the zebrafish otic vesicle requires Otogelin and α-Tectorin
Source: Development. 2015 Mar 15;142(6):1137–45. doi: 10.1242/dev.116632 (PMC4360185; doi:10.1242/dev.116632)
Supplement: Supplementary Material [file supp_142.6.1137_DEV116632supp.pdf]

Supplementary Methods

Behavioural (movement) analysis (see Fig. S1). *eis* and *rst* mutants were sorted from a heterozygous cross at 1 dpf and 3 dpf, respectively. Twelve 5 dpf mutant larvae and 12 siblings were transferred to a 24-well microtitre plate in 500 µl E3 medium (two plates analysed for each mutant). Movement was recorded using the Zebrabox system (Viewpoint, France) using a programme of 4 min with 1 min intervals and speed threshold settings of: slow, <3 mm/s; medium, 3-10 mm/s; fast, >10 mm/s. The total distance covered in fast movement was calculated for each larva and an unpaired Student's *t*-test was used to compare mutant and sibling data (GraphPad Prism).

Mapping the *eis* mutant (see Fig. S3). Genomic SNPs were predicted de novo versus Zv9 and possible deleterious effects were predicted using SNPEff (Cingolani et al., 2012). A critical interval for *eis* was predicted using chromosomal bulk allele frequencies to select the chromosome harbouring the mutation (chromosome 7). Chromosome 7 was then scanned for a region of homozygosity; 7:37031410-49031410 was determined as a critical interval, in which all de novo SNPs were assessed for potential deleterious effects. Finally, known harmless SNPs were subtracted from the candidates; 19 SNPs remained. Of the 19 SNPs, 18 were predicted to cause nonsynonymous amino acid exchanges and one SNP (40879857.A/T) disrupted the splice donor site of exon 28 of *otog*, which we selected for validation.

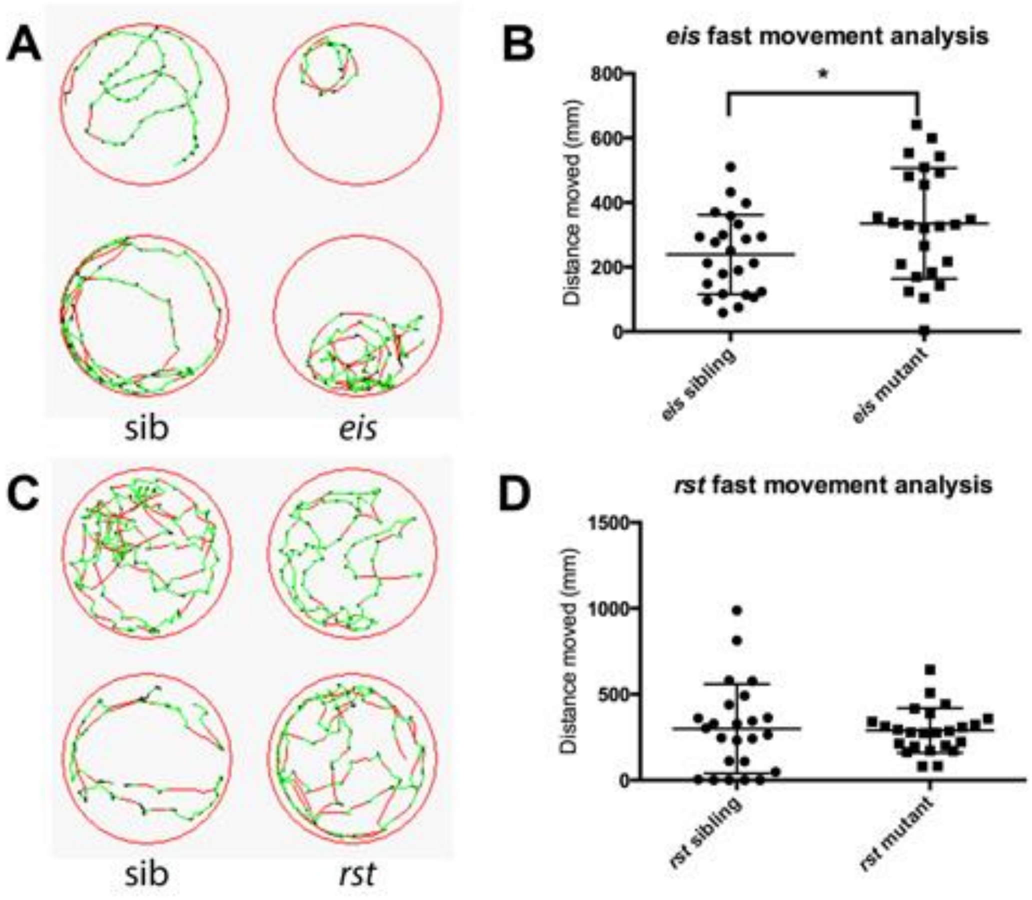

Stooke-Vaughan et al. Supplementary Fig. S1

Fig. S1. Movement analysis of *eis* and *rst* mutant larvae. Comparison of movement of 5 dpf mutant *eis* (A,B) and *rst* (C,D) larvae with phenotypically wild-type siblings from the same batch. (A,C) Examples of movement tracking of individual larvae in a 1 min interval. The colour denotes different speeds: black, <3 mm/s; green, between 3 and 10 mm/s; red, >10 mm/s. Representative traces are shown. *eis* mutants often display circling behaviour (9/17 larvae; examples shown) that is rare in wild-type siblings (3/31 larvae). Examples of sibling traces (A) show a characteristic repeating pattern of green followed by a short stretch of black (i.e. start-stop-start-stop); this pattern is not seen in *eis* mutants. By contrast, *rst* mutants (C) had a similar pattern to their wild-type siblings. (B,D) Scatter plot of distance travelled in fast movement (red lines) in a total of four min (n=24). *eis* mutants (B) are mildly hyperactive, covering more distance in fast movement than their wild-type siblings (P=0.0303). *rst* mutants (D) did not show a difference from sibling larvae in the distance covered in fast movement. Error bars indicate mean ± standard deviation. Preliminary data suggest that abnormalities in swimming behaviour consistent with vestibular defects are present in adult homozyote mutants for both *eis* and *rst* (not shown).

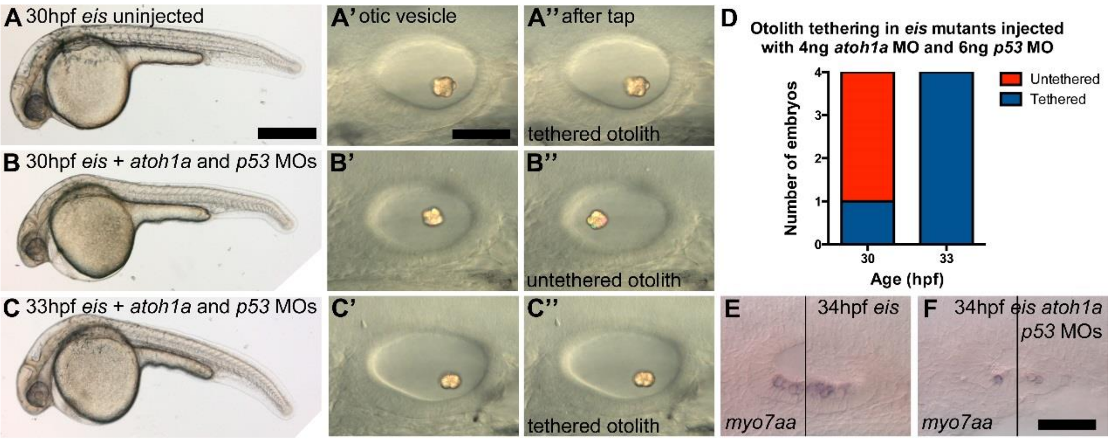

Stooke-Vaughan et al. Supplementary Figure S2

Fig. S2. Otolith tethering in *eis* is independent of *atoh1a* function. (A) 30 hpf uninjected control *eis* mutant embryo. Scale bar: 500 µm. (A′) OV of embryo shown in A, lateral view with anterior to left. A single otolith appears to be tethered to the posterior macula. Scale bar: 50 µm, applies to A′-C″. (A″) The same OV as in A′, after the slide with the embryo mounted on was tapped on the benchtop for ~30 s. The otolith has remained in place and is therefore tethered. (B) 30 hpf *eis* mutant embryo injected with 4 ng *atoh1a* and 6 ng *p53* morpholinos. The embryo shows signs of developmental delay, such as a delay in pigmentation (compare with embryo in A). (B′,B″) OV of embryo shown in B. A single otolith has formed but has not yet tethered, as it is in a different position after the slide with the embryo mounted on was tapped on the benchtop. (C) 33 hpf *eis* mutant embryo injected with 4 ng *atoh1a* and 6 ng *p53* morpholinos. (C′,C″) OV of embryo shown in C. The single otolith is now tethered to the posterior macula. (D) At 30 hpf, one of four *eis* mutants injected with *atoh1a* and *p53* morpholinos had a tethered single otolith (compared with four of four uninjected control *eis* mutant embryos, not shown). At 33 hpf, four of four *eis* mutants injected with *atoh1a* and *p53* morpholinos had a tethered single otolith (compared with two of two uninjected control *eis* mutant embryos, not shown). (E) Lateral view of 34 hpf *eis* uninjected control OV. The region of the anterior macula is shown to the left of the black line; the region of the posterior macula to the right. *myo7aa* expression marks several hair cells in both maculae. (F) Lateral view of the OV of a 34 hpf *eis* mutant injected with *atoh1a* and *p53* morpholinos, showing regions of both the anterior and posterior maculae. Only one anterior and two posterior hair cells are marked by *myo7aa* expression, indicating that no further hair cells have differentiated after the tether cells. Scale bar: 50 µm, applies to E,F.

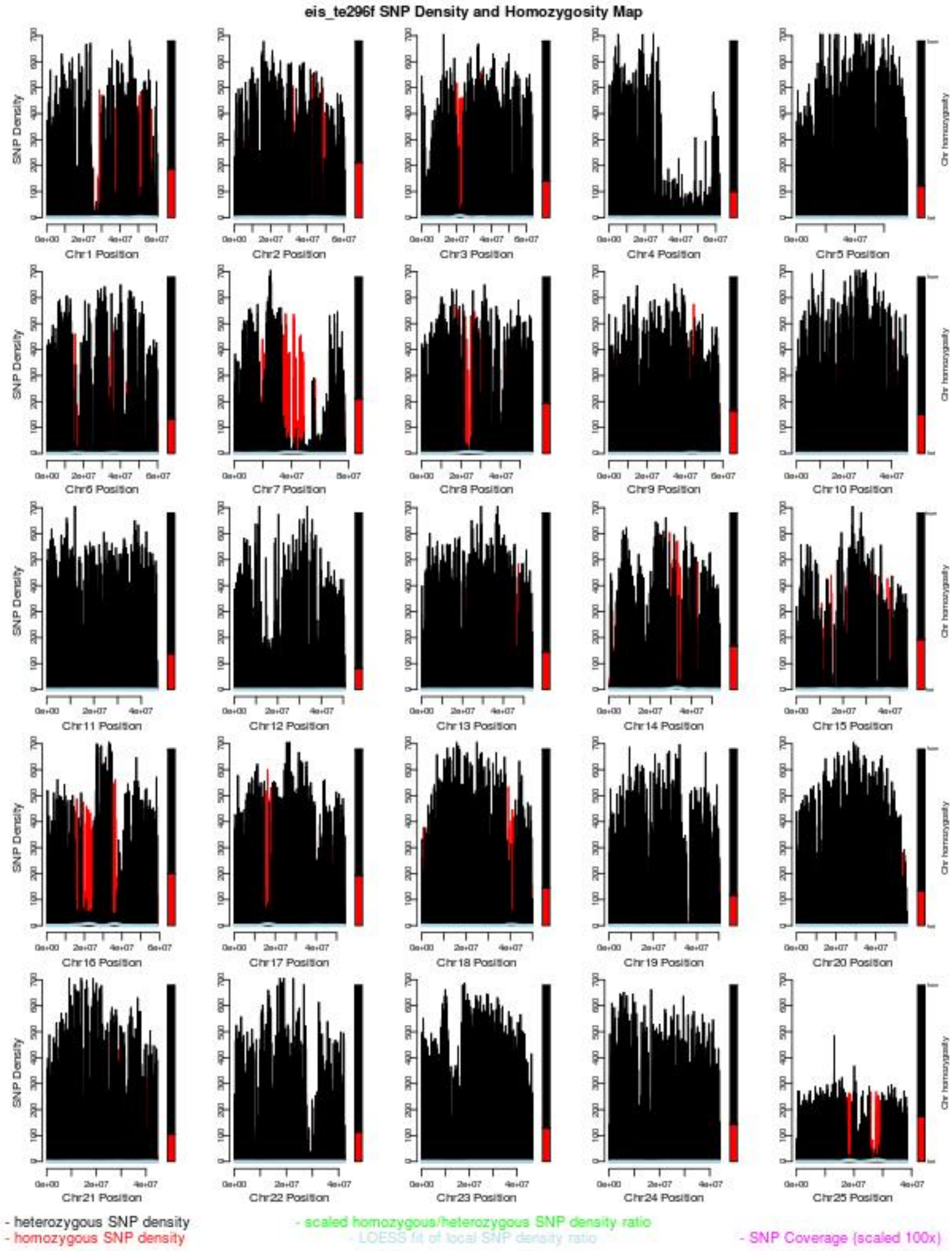

Stooke-Vaughan et al. Supplementary Figure S3A

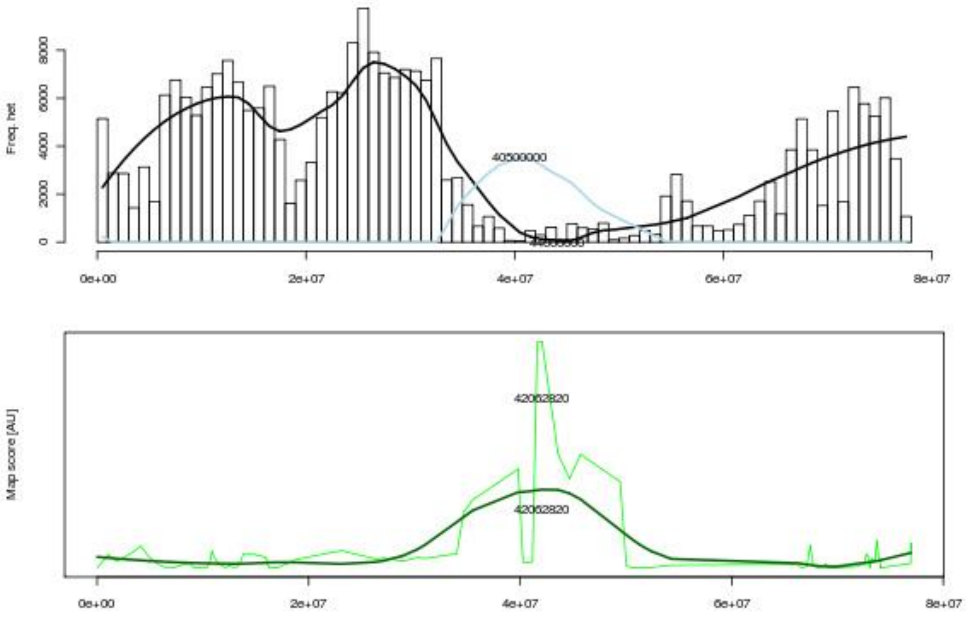

Stooke-Vaughan et al. Supplementary Figure S3B

Fig. S3. Homozygosity mapping and bioinformatic filtering of *eis*^{te296f}. (A) Chromosomal SNP density and homozygosity map of *eis*^{te296f}. Red bars are homozygous SNP counts; black bars are heterozygous SNP counts per 500 kb distance. Split bar graphs next to individual chromosome panels show the degree of homozygosity from 0-1 (red bars) versus heterozygosity (black bars). Chromosome 7 (Chr 7) shows the highest degree of bulk homozygosity and thus is likely to harbour the mutation. Chr 7 also shows a large gap in heterozygosity combined with vast excess of homozygous SNPs, which can be an indication for the position of the critical interval. (B) To estimate the critical interval around *eis*, several statistical predictions for the position of *eis* were generated and averaged. Following one application of the MegaMapper method (Obholzer et al., 2012), the critical interval was set as the predicted position of *eis* ± 6 Mb. Top panel shows a magnification of Chr 7 from A for a valley in the respective SNP density histogram of all detected heterozygous SNPs on Chr 7 and corresponding LOESS fit (black line). The minimum of the fit is indicated with its position (44,000,000). Homozygous-to-heterozygous ratios were fitted for trend recognition using local regression (LOESS; light blue) and its maximum was indicated (40,500,000). One additional statistic was the homozygous-to-heterozygous SNP ratio binned in 1 cM intervals and the maximum of the corresponding fit (42,062,820, green), shown in the lower panel. The average of the resulting values, 43,031,410, was used to position the centre of the critical interval. The homozygous-to-heterozygous ratio fit was used as guidance for a trend in the critical interval, and in this instance closely tracked the actual mutation (40,879,857.A/T) found by in-depth SNP analysis.

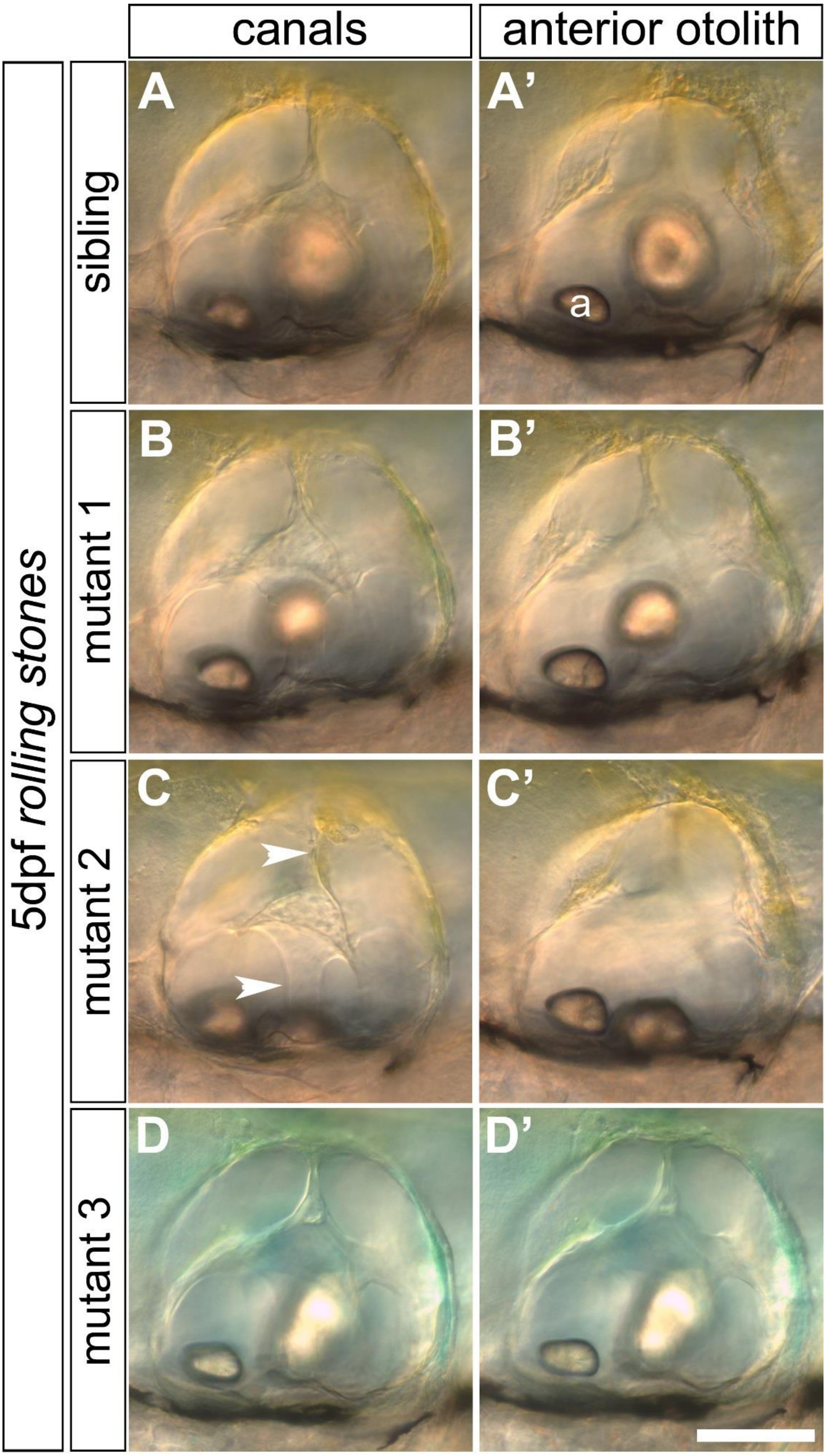

Stooke-Vaughan et al. Supplementary Figure S4

Fig. S4. The anterior otolith and semicircular canal pillars form normally in *rst* mutant embryos. A-D correspond to the OVs shown in B-E of Fig. 3. For each OV, additional focal planes show the semicircular canal pillars and anterior (utricular) otolith. All panels show 5 dpf OVs with anterior to left and dorsal to top. (A,A′) Phenotypically wild-type sibling ear; a, anterior (utricular) otolith. (B,B′) The semicircular canal pillars and anterior otolith appear normal in an *rst* mutant with a detached posterior (saccular) otolith. (C,C′) The canal pillars and anterior otolith appear normal in an *rst* mutant with a posterior otolith stuck on the ventral floor of the OV. Arrowheads in C indicate the dorsolateral septum (top arrowhead) and ventral pillar (bottom arrowhead). (D,D′) The canal pillars and anterior otolith appear normal in an *rst* mutant with a posterior otolith that is misshapen, but still attached to the posterior macula. Scale bar: 100 µm, for all panels.

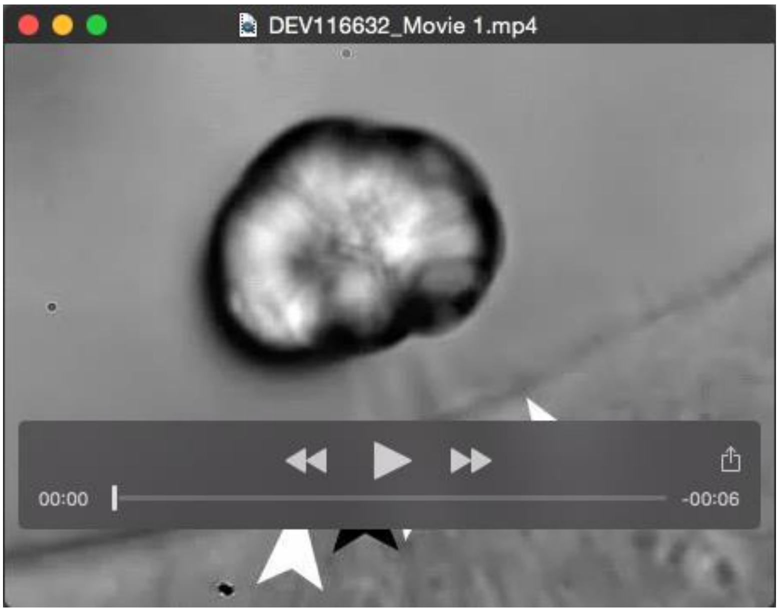

Movie 1. Ciliary motility in a 24 hpf wild-type (AB) ear. Movie of Fig. 1G. Utricular hair cells and anterior otolith of the left ear of a wild-type (AB) embryo in a dorsolateral view with anterior to the right. The movie was recorded at 300 frames per second (fps) and is played back at 15 fps. Two immotile kinocilia tether the otolith (black arrowheads); three motile cilia are near the otolith (white arrowheads).

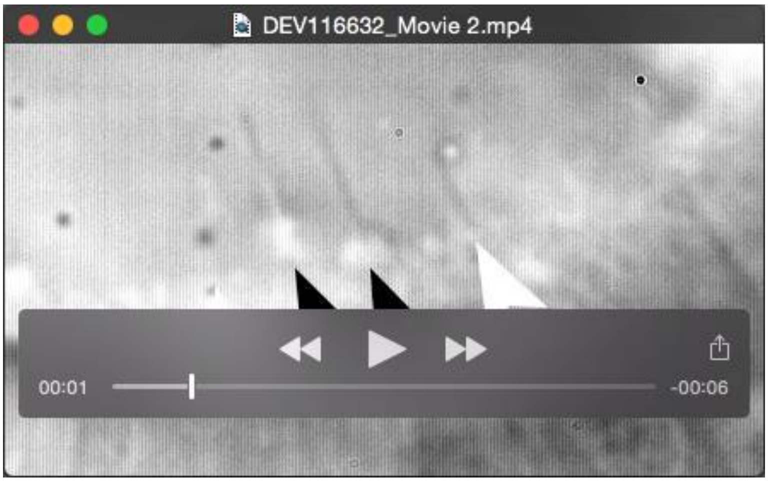

Movie 2. Ciliary motility in a 25 hpf *eis* mutant ear. Movie of Fig. 1H. Utricular tether cells of the left ear of an *eis* mutant embryo in a dorsolateral view with anterior to the right. The movie was recorded at 300 fps and is played back at 15 fps. There is no otolith tethered to the two immotile kinocilia (black arrowheads). Two motile cilia are present near the kinocilia (white arrowheads). More otolith precursor particles are present than in the wild type embryo (compare with Movie 1).

Table S1. Primers

Sequences are listed 5'-3'. Primers were selected using the NCBI Primer Blast tool.

	Forward	Reverse
Primers used to confirm the presence of the SNP in *eis*^{te296f} gDNA		
	TTCTCATCAACACTGGTCAGGTC	TTCTCATCAACACTGGTCAGGTC
Primers used to find the effect of the SNP on *eis*^{te296f} cDNA		
	TTTGCTCAGTGAAGTGTTTCAAGTC	TGGAATCCAAGTGTCTCTATGAAGG
Primers for amplification of exons 35-42/43 of the *otogelin* cDNA		
	TGGGACTGTCCATGTCGTTC	CCGTGATCTCTCCAGTACGC
Primers for amplification of exons 2-27 of *tecta* (Ensembl transcript ENSDART00000082896)		
	CCCTAAGATGGACGATGGCAGTTCC	TTCCCGTCACACAGCTGCACAC
Primer pairs used for sequencing of the large *tecta* clone		
Pair 1	CCCTAAGATGGACGATGGCAGTTCC	GGCAAACCAGGCATTTCCCAGC
Pair 2	GCTTACTACTGCCAGCCCACAC	ACACACACAACGCTGGGAGC
Pair 3	TGAGTGCGGGTGTGAACGTGAC	TAGAGGGCACGGCTCTTCTGGTTC
Pair 4	TGCTACCAACTGCCCTCTACCC	CCGCAACCCACTTGGCAGAAAG
Pair 5	CGACTTTGGTCTGAAGGTGGCG	ACACGGGCGTACCGTTTACC
Pair 6	GAGCACACCCAACCTAACCACC	ATCCTCCCTCTCAAAGCCCAGC
Pair 7	GGCTGAAGTGACCTGTAAGCACGC	TTCCCGTCACACAGCTGCACAC
Primers used for amplification of *tecta* exon 24 from gDNA		
	ACAGTGTCATAAACCTCACACTGCC	GCTGTAACGAGATGGTGTAGCCC
